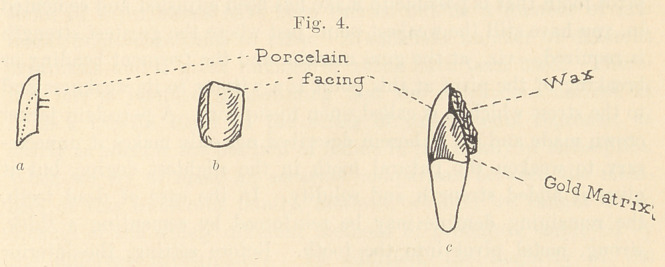# Solbrig’s Porcelain Jacket Crown1Read before the American Dental Society of Europe, at Geneva, Switzerland, April 21, 1905.

**Published:** 1905-10

**Authors:** John H. Spaulding

**Affiliations:** Paris, France


### SOLBRIG'S PORCELAIN JACKET CROWN.[1]

BY JOHN H. SPAULDING, D.D.S., PARIS, FRANCE.

Mr. President and Gentlemen,—Before entering upon a description of the technique used in the operations which are the subject of this paper, I wish to call attention to a few facts which show to whom the profession is indebted for the original idea of such a crown, as well as for the procedure which has resulted in improving and perfecting it, and placing it before the profession as a practical and artistic reality.

In the early Summer of 1903, one of my patients who had very unsightly teeth, owing to extensive erosion, followed by dark brown discoloration, asked me if there was not some way of re-enamelling them. I told her that I did not know of any. Just at this time, I read the article in the *Dental Cosmos,* June, 1903, written by

---

[1] Read before the American Dental Society of Europe, at Geneva, Switzerland, April 21, 1905.

Dr. C. H. Land, of Detroit, in which he speaks of "enamelled caps or jacket crowns." This article interested me so much that I called the attention of my assistant to it. I left in July for America, and promised myself a visit to Dr. Land to inspect his method personally, but was prevented from seeing him.

Upon my return home in September, 1903, my assistant placed in my hands three of the most beautiful, all porcelain jacket crowns which it is possible to imagine,—the result of his experiments during the summer. I was simply delighted with the beauty and natural appearance of these crowns, and we immediately put to a practical use this most artistic invention. I wish to give credit to Dr. C. H. Land for the original suggestion, of which it is the evolution and perfectionment. Judging from the facts related in an article by Dr. Henry W. Gillett, D.M.D., of New York, in the INTERNATIONAL DENTAL JOURNAL for May, 1904, under the title "Spalding's Porcelain Jacket Crown," we have here an undoubted case of coincident and simultaneous invention on the part of Dr. Edward B. Spalding, of Detroit, and Dr. Oscar Solbrig, of Paris, and I believe it to be the most artistic, as well as practical, of anything which has ever been given to our profession, excepting, perhaps, porcelain enamel inlays.

I believe, further, that they will ultimately almost entirely take the place of pivot crowns in any of the six anterior teeth, because the bulk of the natural tooth just where the strain is greatest—at the gum line—does not have to be cut away, the entire strength of the tooth being thus retained, and this is augmented by the porcelain crown.

We have twelve of these crowns doing solid service for many months, some as long as one year and seven months, and we have the greatest confidence in their long durability.

Dr. Solbrig's procedure originated entirely with himself, having for its point of departure nothing but the somewhat vague suggestions contained in the article by Dr. Land, above referred to, and, while arriving at the same practical result as Dr. Edward B. Spalding's, is quite different, and would appear to give a more absolutely exact and certain result with the least trouble to both patient and operator.

Dr. Solbrig makes no shoulder at or under the gum margin, but first removes all existing natural enamel and gives the tooth a conical shape and shortens it as much as possible if living, as much as prudent if dead. (See Fig. 1, *a*, showing tooth with enamel; *a*, *b*, and *c* showing side and front views of prepared stump.)

Fig. 1.

An ordinary plate tooth (we use English teeth which permit of shaping and polishing at will) is then ground out concave on the under side, making a fine, almost feather-edge, where it slips under the gum margin and is exactly fitted to the stump at this point. This we will call the porcelain facing or veneer.[1] A strip of rolled cohesive gold-foil No. 40, the width of which corresponds to the length of the stump, is then placed around the latter and slipped as high as possible under the gum and held in position laterally by pieces of spunk wedged against the adjoining teeth. (See Fig. 2.) This band is then folded with pliers both on the

Fig. 2.

palatal and labial sides (see Fig. 3, *a*), drawing the gold as tightly as possible to the contour of the stump. This is now lifted off the stump and annealed, and the excess of gold where folded is then cut away with scissors, which welds the gold at these points (front

[1] Porcelain veneers are now produced by the manufacturers of porcelain teeth in America, and I hope Ash & Sons will also do so very soon.

and back), leaving a conical cap, or matrix, of gold (Fig. 3, *b*). This cap is now replaced on the stump and reburnished over its entire surface. A little beeswax is then placed on the labial surface of this gold matrix, as well as in the concave of the porcelain

Fig. 3.

facing; also a small softened piece against the lingual side of the matrix; the facing is then placed in position and pressed home, if necessary, with a warm spatula. (See Fig. 4, *a*, *b*, and *c*.) When cool, the whole is removed from the stump and invested in

Fig. 4.

powdered asbestos paste. It will be noticed that the relative positions of porcelain facing and gold matrix, as well as the adjustment on the stump are absolutely exact and the investment in powdered asbestos maintains these conditions, *and also enters the matrix, taking the place of the stump itself.* After drying, the wax is removed by warming and, finally, burning out, and its place between the facing and the matrix is filled with porcelain, which is then fused, thus uniting the facing and matrix.

Additions of porcelain and repeated fusings are made until this space is entirely filled. The investment is then entirely removed, *except that part which enters the matrix,* and which represents the stump of the tooth. It is very essential to retain this part of the investment until the crown is entirely finished, as it prevents any possible change in the shape of the matrix or gold cap during manipulation. Subsequent additions of porcelain and fusings are then made until the lateral and lingual aspects of the crown are covered and the crown is complete. There is no necessity to have the patient return for reburnishing of the gold matrix.

When the bakings are finished, the gold matrix is stripped out, leaving a porcelain cap exactly the same shape and form as would be the natural enamel were it possible to lift it off from the dentine, but it is much thicker and stronger, except at the margins where it is made exactly to represent and replace the natural enamel. There is no shoulder to fit, therefore no joint, and in the case of recession of the gum the tooth would not be more unsightly than a natural tooth under the same condition.

I wish to call your especial attention to the great value this method has, apart from its artistic appearance, over the ordinary pivot tooth of any system in the matter of strength. When a canine or incisor is cut off even with the gum, and the strongest pivot tooth that is possible to make has been adjusted and cemented in, you have still the weakest point just where the greatest strength is required,—viz., at the gum margin, and the frequent bending or breaking of the pivot at this point is a witness to its weakness and to the stress which it is called upon to support. A porcelain jacket crown made and set as herein described not only makes it unnecessary to weaken the natural tooth in the slightest degree, but it gives it added strength and solidity. In the case of dead teeth, the remaining dentine may be reinforced by cementing a thick, strong, metal pivot into the tooth. Before setting, the interior of the jacket crown should be roughened or etched with hydrofluoric acid, thus giving a surface much better adapted for the attachment of the cement which insures the maximum of strength between the porcelain and the stump. For setting, we give the preference to Harvard cement, as it is very tenacious and becomes exceedingly hard with time.

We use Dr. Jenkins's new porcelain enamel, or prosthetic porcelain which fuses just under the fusing point of pure gold, and is exceedingly strong and fine grained. It unites perfectly with the English teeth and can be ground and repolished as circumstances and convenience require. We consider it the *ne plus ultra* of strength, reliability, and convenience of manipulation.

## Figures and Tables

**Fig. 1. f1:**
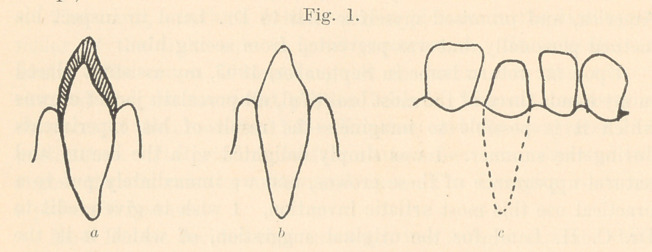


**Fig. 2. f2:**
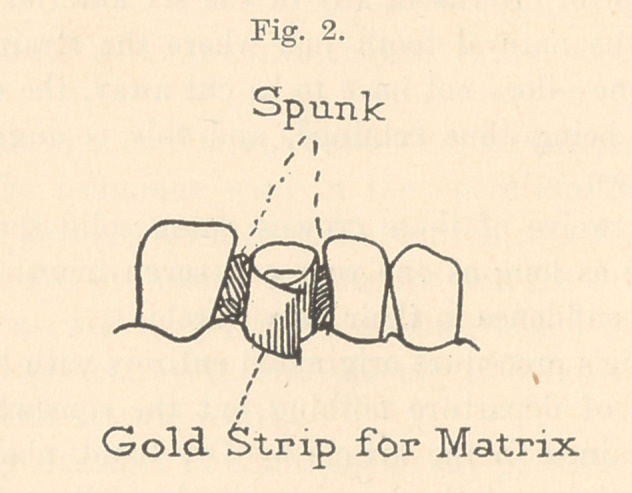


**Fig. 3. f3:**
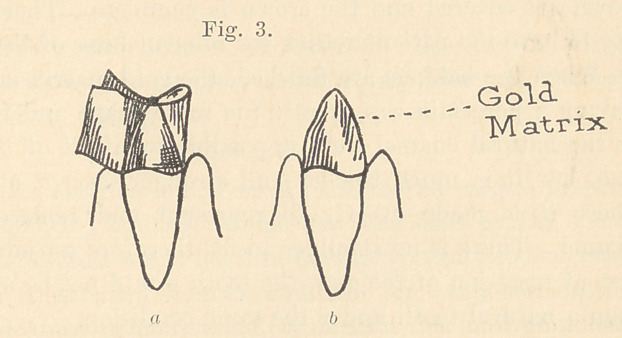


**Fig. 4. f4:**